# Molecule Dynamics Simulation of the Effect of Oxidative Aging on Properties of Nitrile Rubber

**DOI:** 10.3390/polym14020226

**Published:** 2022-01-06

**Authors:** Jinsong Yang, Weitao Lou

**Affiliations:** 1 School of Traffic and Transportation Engineering, Central South University, Changsha 410075, China; yangjs@csu.edu.cn; 2 Graduate School of China Academy of Engineering Physics, Beijing 100193, China

**Keywords:** nitrile rubber, oxidative aging, molecule dynamics simulation, hydroxyl groups, carbonyl groups

## Abstract

The effects of oxidative aging on the static and dynamic properties of nitrile rubber at the molecular scale were investigated by molecular dynamics simulation. The aged nitrile rubber models were constructed by introducing hydroxyl groups and carbonyl groups into rubber molecular chains to mimic oxidative aging. The static and dynamic properties of the unaged and aged nitrile rubber under different conditions were evaluated by mean square displacement, self-diffusion coefficients, hydrogen bond, fractional free volume, radial distribution function, cohesive energy density and solubility parameter. The results show that the elevated temperature intensified significantly the mobility of rubber molecular chains and fractional free volume, while the compressive strain displayed the opposite effect resulting in packing and rearrangement of rubber chains. The introduction of hydroxyl groups and carbonyl groups enhanced the polarity, intermolecular interactions, the volume and rigidity of molecular chains, implying weaker mobility of molecular chains as compared to unaged models. The compressive strain and oxidative aging both decreased the fractional free volume, which inhibited gaseous and liquid diffusion into the rubber materials, and slowed down the oxidative aging rate. This study provides insights to better understand the effect of molecular changes due to oxidative aging on the structural and dynamic properties of rubber materials at the molecular level.

## 1. Introduction

Nitrile rubber (NBR) has been widely used as seals in sealing structures of hydraulic systems [1], due to its excellent oil resistance and mechanical properties. However, under practical service conditions, the rubber seals are often subjected to thermal cycles, oxygen, chemical media and mechanical stress leading inevitably to deterioration in chemical structures and mechanical properties [2,3,4,5]. With increasing service time, the rubber seals gradually lose elasticity which can lead to sealing failures and consequent leakage of gases and liquid [6,7,8,9].

The degradation mechanism of NBR under high temperature, chemical medium, radiation and stress has been widely studied for decades [10,11,12,13,14]. These studies indicate that the degradation mechanism of NBR includes physical reactions and chemical reactions, leading to deterioration in physical and mechanical properties [15]. The physical reactions consist of migration and volatilization of small molecular additives, creep and relaxation of rubber molecular chains [16,17]. The chemical reactions are made up of oxidation reactions such as crosslinking reactions, chain scission reactions and formation of oxidation products, which are mainly responsible for the degradation of NBR [14,18,19,20]. During the oxidative aging process, oxidation reactions occur leading to the formation of oxidation products such as hydroxyl groups, carbonyl groups and hyperoxide [21]. The formation of oxygen-containing groups enhances the polarity of rubber molecular chains, which seriously affects intermolecular interaction between rubber chains, energy distribution, and the mobility and flexibility of rubber chains, resulting in obvious changes in physical and chemical properties of NBR [22,23]. Moreover, the formation of polar groups in rubber chains may have positive or negative effects on diffusion behaviors of oxygen and other chemical species into the rubber materials, which may promote or inhibit degradation rates [24]. Many researchers focus on understanding the changes in rubber materials at the molecular level in an indirect or qualitative way, especially for oxidation products, associated with physical or mechanical properties by advanced experimental characterization methods, such as Fourier transform infrared spectroscopy (FTIR), X-ray photoelectron spectroscopy (XPS) and nuclear magnetic resonance spectroscopy (NMR) [25,26,27]. However, the molecular changes caused by oxidative aging on the static and dynamic characteristics of NBR at the molecular level still remain relatively unexplored.

In recent years, molecular dynamics (MD) simulation has been employed as an effective theoretical tool to comprehend quantitatively the structure–performance relationships of polymer materials at the molecular scale, especially in modification of polymers and diffusion of small molecules in polymers [28,29]. Although the molecular dynamics simulation methods were preliminarily applied to estimate the thermal oxidative aging mechanism of rubber materials [30,31,32], the chemical reactions such as oxidation products during the aging process are not fully considered by researchers. For example, Wang et al. [30] found that rubber at low compressive strain possesses high fractional free volume, molecular chain movement, and ozone permeability by molecular dynamics simulation. Zhi et al. [31] studied the heterogeneous oxidative aging and viscoelastic performance of rubber based on multi-scale simulation. The molecular dynamics simulation was used to investigate the permeability of oxygen in natural rubber. Hence, the influence of oxidative aging on the structural and dynamic properties of nitrile rubber requires further investigation.

In our previous studies, degradation behavior and the mechanism of rubber seals under actual service conditions such as elevated temperature, hydraulic oil and compressive strain were systematically investigated by accelerated tests [33,34]. It was found that the chemical reactions during the aging process, especially the oxidation products, presented significant effects on degradation in physical and mechanical properties of rubber seals. Thus, this work focuses on the influence of the oxidation products (hydroxyl group and carbonyl group) on the static and dynamic properties of NBR by using molecular dynamics simulation. Additionally, the aged NBR models were constructed on the basis of the oxidative rubber chains modified by oxidation products. After the molecular dynamics simulations, the influences of oxidative aging of rubber molecular chains on static and dynamic performances of NBR at different temperatures under uncompressed and compressed state at molecular scale were investigated by analyzing the mean square displacement (MSD), self-diffusion coefficients, hydrogen bonds, fractional free volume, radial distribution function and cohesive energy density.

## 2. Simulation Models and Methods

**Figure 1:**
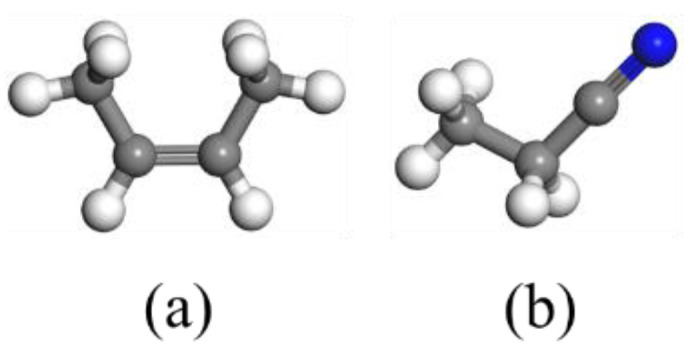
The repeating units of butadiene (**a**) and acrylonitrile (**b**).

**Figure 2:**
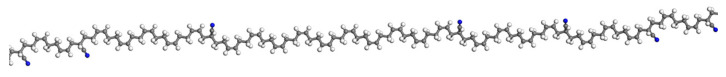
The chemical structure of the nitrile rubber molecule chain.

To study the effects of oxidative aging on nitrile rubber properties at the molecular level, unaged and aged nitrile rubber models were constructed by using the Forcite and Amorphous cell modules of Materials Studio 8.0 softwares, respectively. The COMPASS (condensed-phase optimized molecule potentials for atomistic simulation studies) force field was used for describing molecule interactions and intermolecular potential. The COMPASS force field is widely suitable for most common organics, small inorganic molecules and polymers, and can accurately predict the structural and thermophysical condensed phase properties of the related materials under a wide range of temperature and pressure conditions. The electrostatic and van der Waals forces were calculated by using the atom-based summation method. The Maxwell−Boltzmann profiles were used to set the initial velocities. Firstly, the repeating units of butadiene and acrylonitrile were constructed, respectively, shown in Figure 1. Then, the butadiene and acrylonitrile were copolymerized to form a nitrile rubber molecule chain with an acrylonitrile content of 35%, containing 50 repeating units. The chemical structures of the nitrile rubber molecule chain is shown in Figure 2. Secondly, an amorphous cell of the unaged NBR model was constructed with five molecule chains.

**Figure 3:**
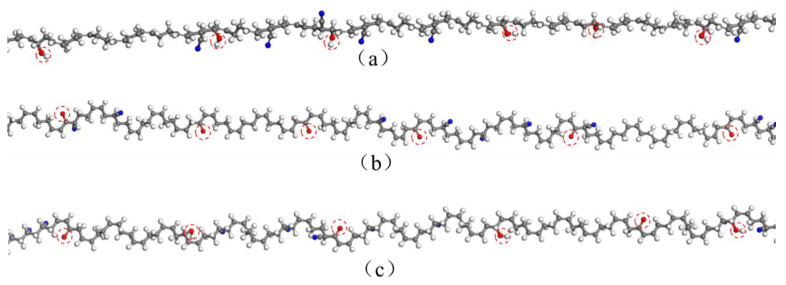
The aged rubber molecule chains modified by oxidation products: (**a**) hydroxyl groups; (**b**) carbonyl groups; (**c**) hydroxyl groups and carbonyl groups.

**Figure 4:**
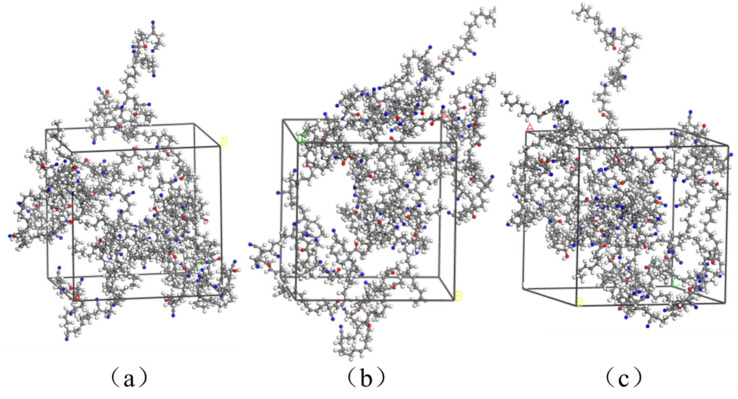
The aged nitrile rubber (NBR) model: (**a**) aged NBR model modified by hydroxyl groups (OH-NBR); (**b**) aged NBR model modified by carbonyl groups (CO-NBR); (**c**) aged NBR model modified by hydroxyl groups and carbonyl groups (OH-CO-NBR).

Previous studies indicated that the oxidation of rubber chains was mainly responsible for the performance degradation, accompanied by the formation of oxygen-containing functional groups [11,16,22,33,34,35,36,37]. The hydroxyl group and carbonyl group have been identified as major functional groups, which were formed during the oxidative aging process. The formation of these oxidation products can lead to changes in the molecular structure of the polymer matrix, which further results in degradation of physical and mechanical properties of rubber materials. Therefore, this study adopted the introduction of the hydroxyl groups and carbonyl groups in the molecule chains to modify the rubber chains in specified locations, representing oxidative aging effects. Figure 3 presents the aged rubber molecule chains modified by hydroxyl groups and carbonyl groups, respectively. Then, three types of aged NBR model were constructed, respectively, shown in Figure 4. In this study, the unaged NBR model is defined as “NBR”. The aged NBR model modified by hydroxyl groups is defined as “OH-NBR”. The aged NBR model modified by carbonyl groups is defined as “CO-NBR”. The aged NBR model modified by hydroxyl groups and carbonyl groups together is defined as “OH-CO-NBR”. The degree of polymerization values, DP, the total number of chains, ***N*_chain_**, the total number of atoms, *N*_atom_, and cell lengths, for the constructed models are given in Table 1. Although the methods employed in this work can be used to investigate the effects of hydroxyl groups and carbonyl groups on nitrile rubber properties, it is still impossible to directly simulate the real thermal-oxidative aging including oxidation products, crosslinking, and chain scission. Coupled effects of the oxidation products, crosslinking, and chain scission should be considered in further studies by introducing aging information.

In our previous publications [33,35], the effects of elevated temperature and compression deformation on physical and chemical properties of nitrile rubber were investigated. This study focuses on how the elevated temperature and compression deformation influence the molecular structure of the aged NBR. A 30% compressive strain was respectively applied to the unaged and aged NBR models based on the actual compression state in the experimental study [35]. The compression deformation was applied along the Z-direction. Additionally, the X-direction and Y-direction were constrained to change shape and size of NBR models.

For the unaged and aged NBR models, the smart minimizer algorithm was applied to minimize the amorphous cells at 298.15 K for 1,000,000 steps, until a convergence value of 1.0 × 10^−5^ Kcal mol^−1^ Å^−1^ was reached. Then, the cells were annealed at 0.1 MPa from 600 K to 300 k for 200 ps (picosecond). After that, 200 ps of NPT (constant number of particles, pressure, and temperature) simulation was conducted at 300 K and 2 GPa, 1 GPa, 0.5 GPa to further relax the rubber chains, respectively. Then, 500 ps of NVT (constant number of particles, volume, and temperature) simulation was carried out at 300 K. Subsequently, 500 ps of NPT simulation was conducted at 300 K and 101 KPa to obtain the stable structure. The simulated densities of the unaged NBR models were obtained and are given in Table 1. The simulated densities agreed well with the experimental values ($\left|\rho_{MD}-\rho_{Exp}\right|<$ 0.1 g/cm^3^), which proved the reliability of models [28]. The temperature and pressure were controlled by the Andersen thermostat [38] and Berendsen barostat [39], respectively. Finally, a 500 ps NVT product run was carried out at 101 kPa and 298.15 K (25 °C), 343.15 K (70 °C), 363.15 K (90 °C) and 383.15 K (110 °C), respectively, to obtain finally a stable structure and analyze the thermodynamic properties. These temperatures were selected on the basis of our previous experimental study [35]. Then, the radial distribution function (RDF), cohesive energy density (CED), the numbers and types of H-bonds, the fractional free volume (FFV), and radial distribution function (RDF) were estimated by further analysis of the equilibrated cells.

## 3. Results and Discussion

### 3.1. Mean Square Displacement (MSD) and Self-Diffusion Coefficients

**Figure 5:**
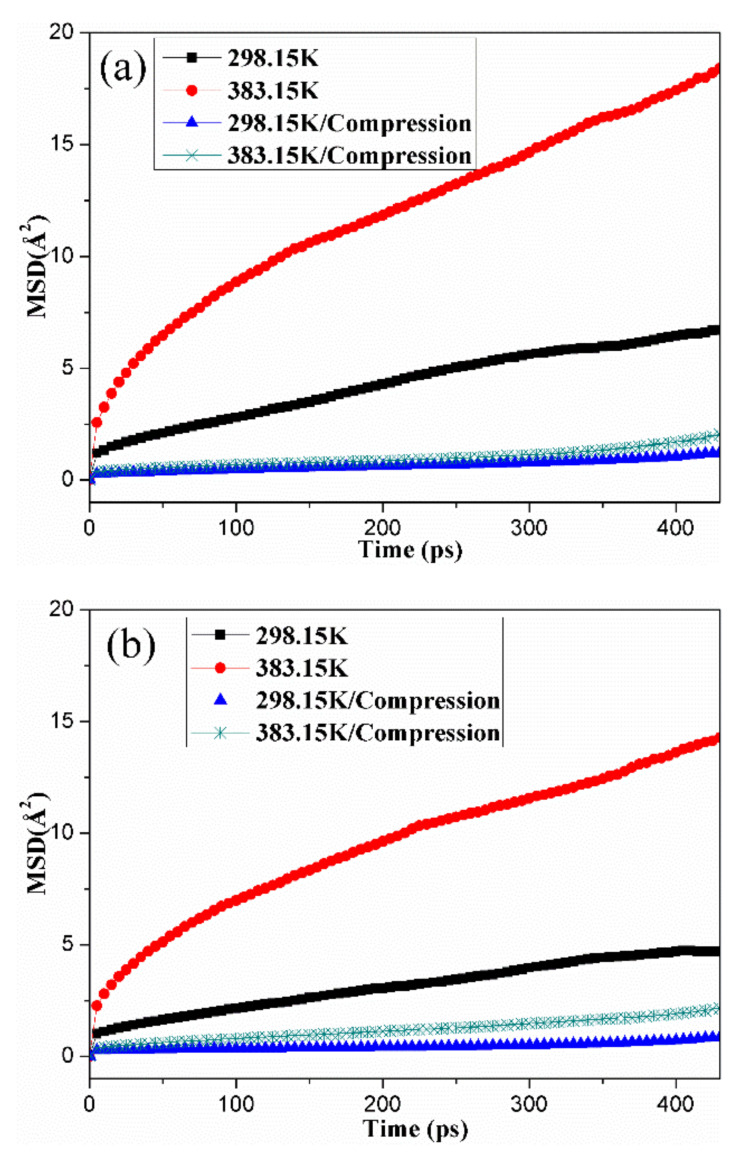

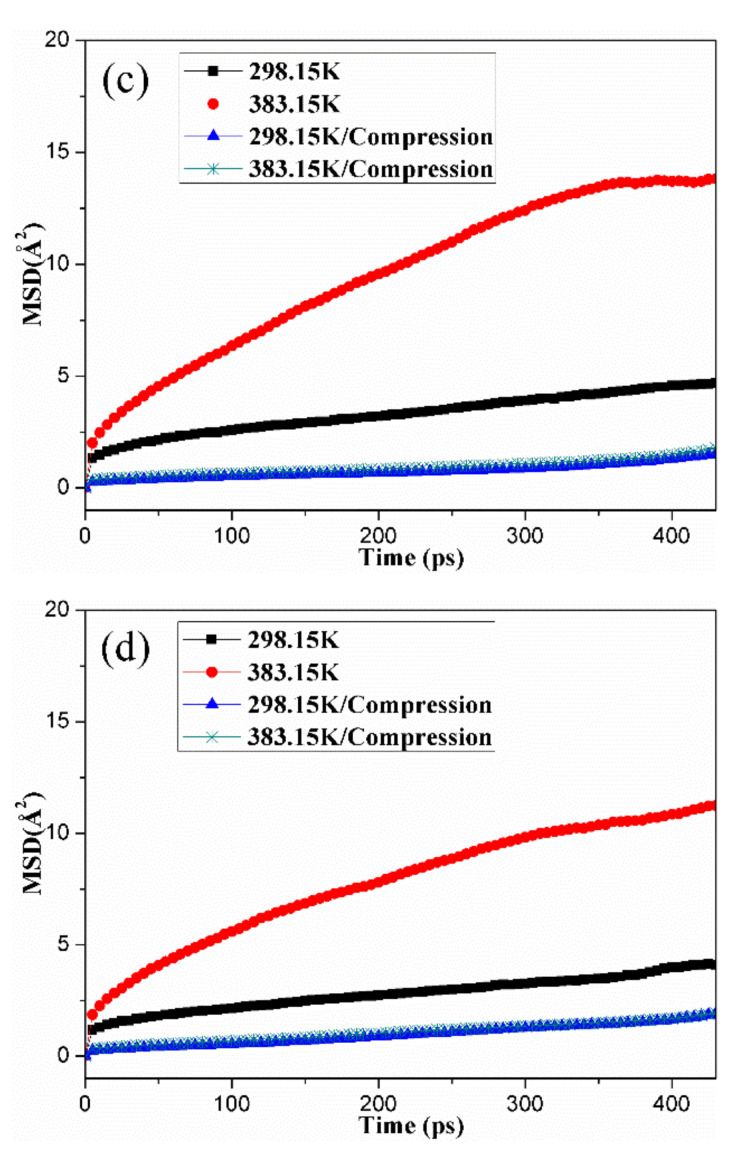
Changes in mean square displacement (MSD) of rubber molecule chains in unaged and aged NBR models under different conditions: (**a**) NBR; (**b**) OH-NBR; (**c**) CO-NBR; (**d**) OH-CO-NBR.

To find out the effects of temperature, compression deformation and oxidative aging on the mobility of nitrile rubber molecule chains, the MSDs of rubber molecule chains in unaged and aged NBR models under different conditions were calculated, as shown in Figure 5. The results show that the MSDs of rubber chains increased with increasing simulation time, which is attributed to the motion of molecule chains. Besides, under the free state, the higher the temperature was, the faster the MSD increased, which indicates that high temperatures could significantly promote the mobility of chains [40,41,42]. Furthermore, the value of MSD under the compression state was obviously lower than that of the chains under the free state, even at higher temperature, implying that mobility of molecule chains was greatly restricted under the compression state. For the unaged and aged NBR models, a similar change tendency in MSD of the uncompressed and compressed samples was observed, and the value of MSD of the molecule chains in unaged NBR models was greater than that in aged NBR models. The MSD of the molecule chains in the OH-CO-NBR model presented the smallest value among the aged NBR models.

To further analyze the influence of hydroxyl groups and carbonyl groups on motion ability of rubber chains, the MSD was used to estimate self-diffusion coefficients by using the Einstein equation [30,39,43,44], which is expressed as:

(1) $$D=\frac{1}{6N}\underset{t\to \infty }{\lim}\frac{d}{dt}\sum_{i=1}^{N}\langle {\left|r_{i}\left(t+t_{0}\right)-r_{i}\left(t_{0}\right)\right|}^{2}\rangle$$

where *N* is the number of molecules, *D* is the diffusion coefficient of rubber molecule chains, $r_{i}\left(t_{0}\right)$ is the displacement at *t*_0_, $r_{i}\left(t+t_{0}\right)$ is the displacement at *t*_0_ + *t*, and

(2) $$MSD\left(t\right)=\frac{1}{N}\sum_{i=1}^{N}\langle {\left|r_{i}\left(t+t_{0}\right)-r_{i}\left(t_{0}\right)\right|}^{2}\rangle$$

Table 2 shows the self-diffusion coefficients of rubber chains under different conditions. The results indicate that self-diffusion coefficients of unaged and aged NBR chains increased with temperature. Thus, the self-diffusion coefficients of rubber chains in different models conform to the order: NBR > OH-NBR > CO-NBR > OH-CO-NBR, which indicates that the mobility of the unaged rubber molecular chains was stronger than that of the aged rubber chains.

Due to the addition of hydroxyl groups and carbonyl groups to the molecular chains, the energy and volume space required for the movement of the molecular chains is greater than that of the unaged rubber molecular chains, leading to the decrease in mobility. The decrease in MSD and self-diffusion coefficient of rubber molecular chains caused by oxidation aging were mainly due to the increase in volume and rigidity of rubber molecular chains.

Furthermore, the MSDs and self-diffusion coefficients of rubber molecular chains under the compression state displayed lower value compared to those in the uncompressed state. However, the MSDs of the compressed rubber molecular chains showed no obvious changes at low and high temperature, implying that the application of compressive strain resulted in the packing and rearrangement of molecular chains, and significantly limited the mobility of rubber molecular chains.

### 3.2. Hydrogen Bond

During the oxidative aging process, the oxidation functional groups that have strong polarity were formed in the rubber molecular chains. In this study, the hydroxyl groups and carbonyl groups were introduced in the rubber molecular chain to represent the oxidative aging of rubber molecular chain. The introduction of the polar groups contributed to the formation of strong polar interaction including non-bonded interaction and hydrogen bonds among the oxidized rubber chains, which affected the chain dynamics and structure properties of the rubber [29]. Therefore, it is necessary to analyze the influence of the type and number of hydrogen bonds in the rubber models.

**Figure 6:**
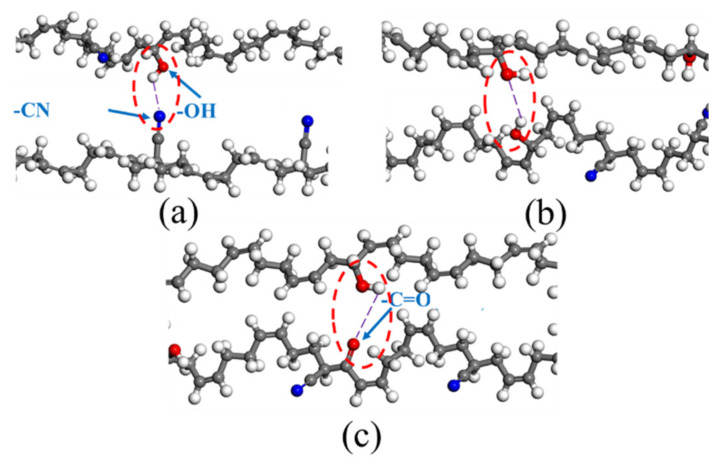
The types of H-bond in the aged rubber model: (**a**) H-bond _(A)_ or O–H...N; (**b**) H-bond _(B)_ or O–H...O (–OH); (**c**) H-bond _(C)_ or O–H...O (–CO).

**Figure 7:**
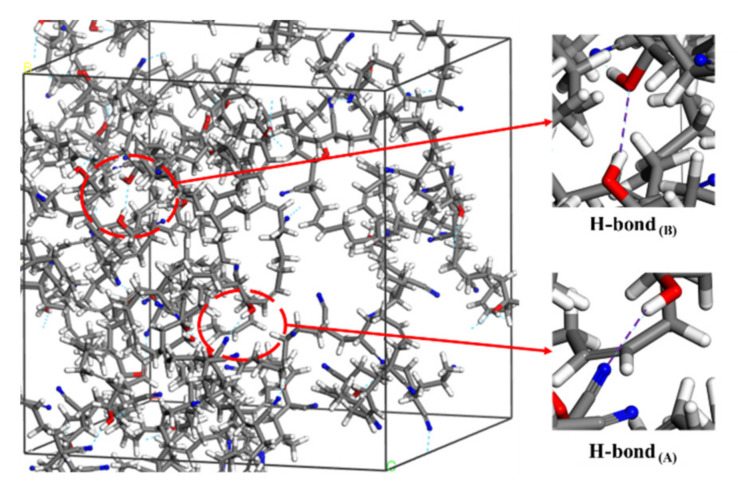
Two types of H-bond in OH-NBR model.

**Figure 8:**
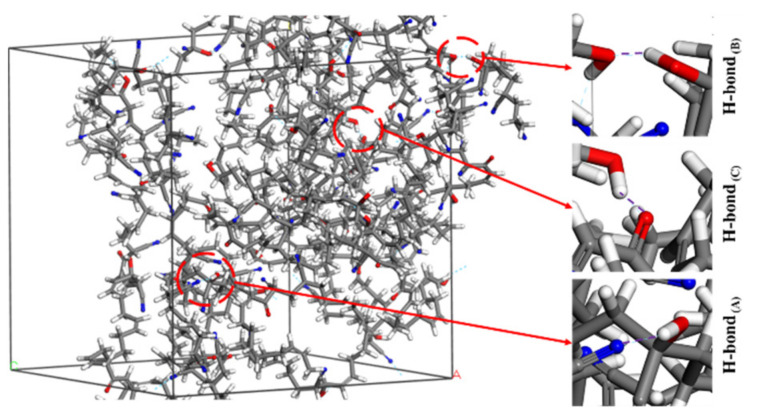
Three types of H-bond in OH-CO-NBR model.

Figure 6 shows the types of H-bond in the aged rubber model. The first H-bond _(A)_ or O–H...N (Figure 6a), was formed between the hydroxyl group and the nitrile group. The second H-bond _(B)_ or O–H...O (Figure 6b), was formed between the hydroxyl group and the hydroxyl group. The third H-bond _(C)_ or O–H...O (Figure 6c), was formed between the hydroxyl group and the carbonyl group. By further analysis, it was found that the H-bonds occurred only in the OH-NBR model and OH-CO-NBR model, shown in Figure 7 and Figure 8. Meanwhile, Table 3 shows the types and number of H-bonds in aged NBR models. The results show that the number of H-bonds _(A)_ in the uncompressed OH-NBR model and OH-CO-NBR model decreased with increasing temperature, but presented the opposite trend in the compressed state. Additionally, the number of H-bonds _(B)_ in the OH-NBR model and OH-CO-NBR model displayed no obvious changes with temperature. The H-bond _(C)_ only existed in OH-CO-NBR model. Moreover, the number of H-bonds _(C)_ in the uncompressed OH-CO-NBR model showed a slight increase with temperature. These phenomena illustrate that the elevated temperature destroyed the H-bond _(A)_ and H-bond _(B)_ in the uncompressed state. This is mainly because the elevated temperature promoted the mobility of molecular chains and increased the chain space, until the intermolecular distance could no longer meet the conditions to form the hydrogen bond. However, the high temperature promoted the formation of the H-bond _(A)_ under the compression state due to the sufficient energy supplied by elevated temperature. However, at 298.15 K, the number of H-bonds _(A)_ in the compressed state was less than that in the uncompressed state. The results demonstrate that compressive strain destroyed the hydrogen bonds and limited the formation of new hydrogen bond, due to the lower mobility of rubber molecular chains under the coupled effect of lower temperature and compressive strain, which was explained by the results of the MSD and self-diffusion coefficient of rubber molecular chains. However, at 383.15 K, the changes in the number of H-bonds _(A)_ showed an adverse trend, implying that the coupled influence of compressive strain and elevated temperature facilitates the formation of H-bond _(A)_. Nevertheless, the results of the MSD and self-diffusion coefficient of rubber molecular chains at 383.15 K in the compressed state displayed a lower mobility of rubber molecular chains. Thus, the increase in the number of H-bonds _(A)_ was attributed to the fast motion of local segments and side groups caused by the high temperature. The formation of H-bonds strengthened the interaction force between molecular chains, and further restricted the movement of rubber molecular chains.

### 3.3. Fractional Free Volume (FFV)

Fractional free volume (FFV) can be defined as the fraction of the volume not occupied by the polymer, which is often used to estimate the available space for free movement of rubber chain and the efficiency of rubber chain packing. The FFV can be calculated by the following equation:

(3) $$FFV=1-\frac{V_{0}}{V_{S}}$$

where the occupied volume *V*_0_ = 1.3 *V*_w_, *V*_w_ is the van der Waals’s volume, and vs. is the specific volume.

**Figure 9:**
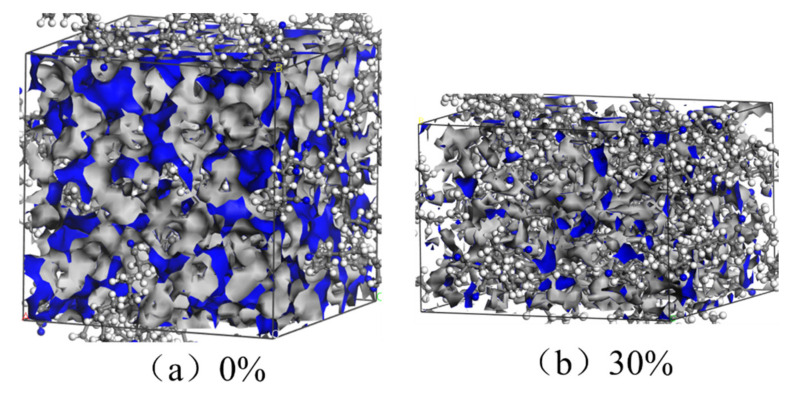
The Connolly volume morphology of NBR model in the uncompressed (**a**) and compressed (**b**) states, respectively (the blue and gray regions represent the free volume and the occupied volume, respectively).

**Figure 10:**
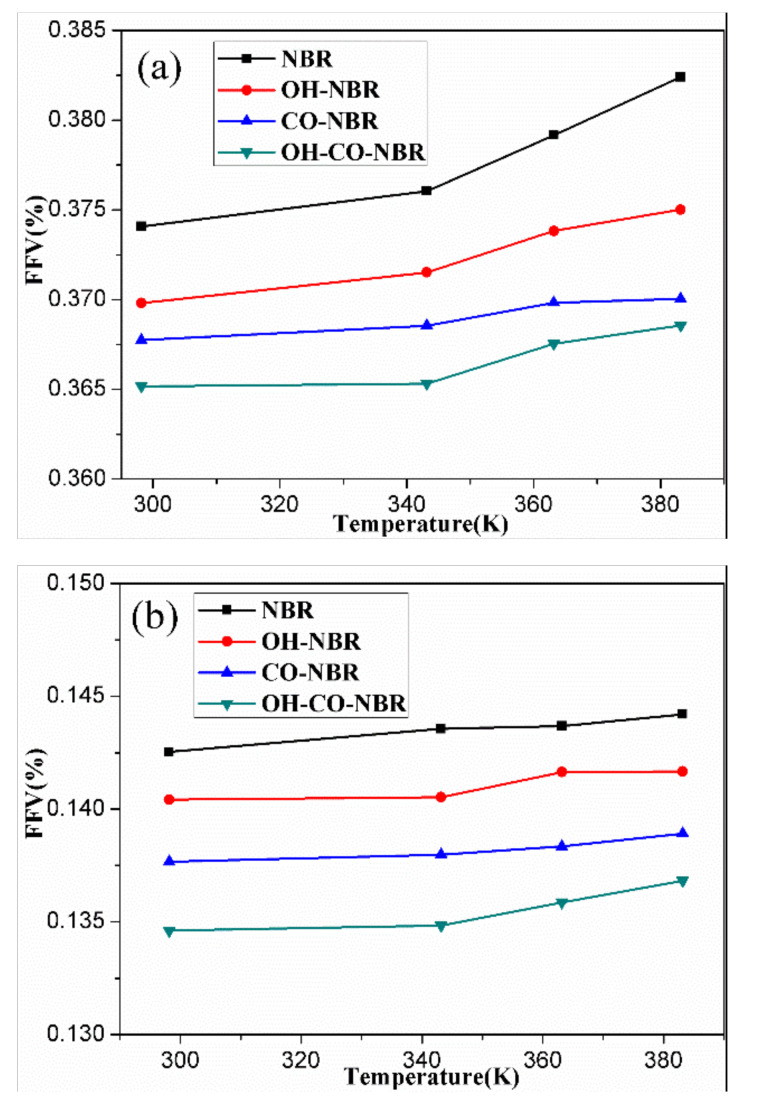
The fractional free volume (FFV) of the unaged and aged NBR models in the uncompressed (**a**) and compressed (**b**) state.

Figure 9 shows the Connolly volume morphology in the uncompressed and compressed states. The blue and gray regions represent the free volume and the occupied volume, respectively. Figure 10 presents the FFV of the unaged and aged NBR models in the uncompressed and compressed states. The FFV of rubber models all increased with increase in temperature, and the free volume fraction of rubber in the uncompressed state presented a bigger value than that in the compressed state. Furthermore, the FFV of the unaged NBR models was greater than that of the aged NBR models. Additionally, the FFV value of the aged models conforms to the order: FFV_OH-NBR_ > FFV_CO-NBR_ > FFV_OH-CO-NBR_.

The volume of rubber models is mainly composed of the volume of the rubber chain and the free volume caused by thermal movement of rubber chain. With increase of temperature, the conformational entropy increased and chain segments motioned significantly, leading to an increase of FFV [30]. Additionally, the application of compressive strain limited the thermal movement of the rubber molecular chains, resulting in efficient molecular packing and a decrease of FFV. When the hydroxyl groups and carbonyl groups were introduced in the rubber molecular chains, these polar groups contributed to the formation of strong polar interactions among molecule chains. Moreover, the introduction of the polar groups increased the rigidity of rubber chains, and promoted tight packing of rubber molecular chains, which led to decrease in FFV. These results indicate that the compressive strain and oxidation functional groups both have a negative influence on the changes of FFV.

### 3.4. Radial Distribution Function (RDF)

The radial distribution function (RDF) is defined as the probability of finding another atom at a distance from a specific atom, which can be used to estimate the interactions between the components and static properties of a rubber model. The RDF can be calculated by the following equation [29]:

(4) $$g(r)=\frac{1}{\rho_{B}}\frac{1}{N_{A}}\sum_{i}^{N_{B}}\sum_{j}^{N_{A}}\frac{\delta \left(r_{ij}-r\right)}{4\pi r^{2}}$$

where *r* is the distance, *r_ij_* is the coordinate of the atom; *N_A_* and *N_B_* are the numbers of atom A and B; *ρ_B_* is the average density of the atom B at a given distance *r* as a center of atom A.

**Figure 11:**
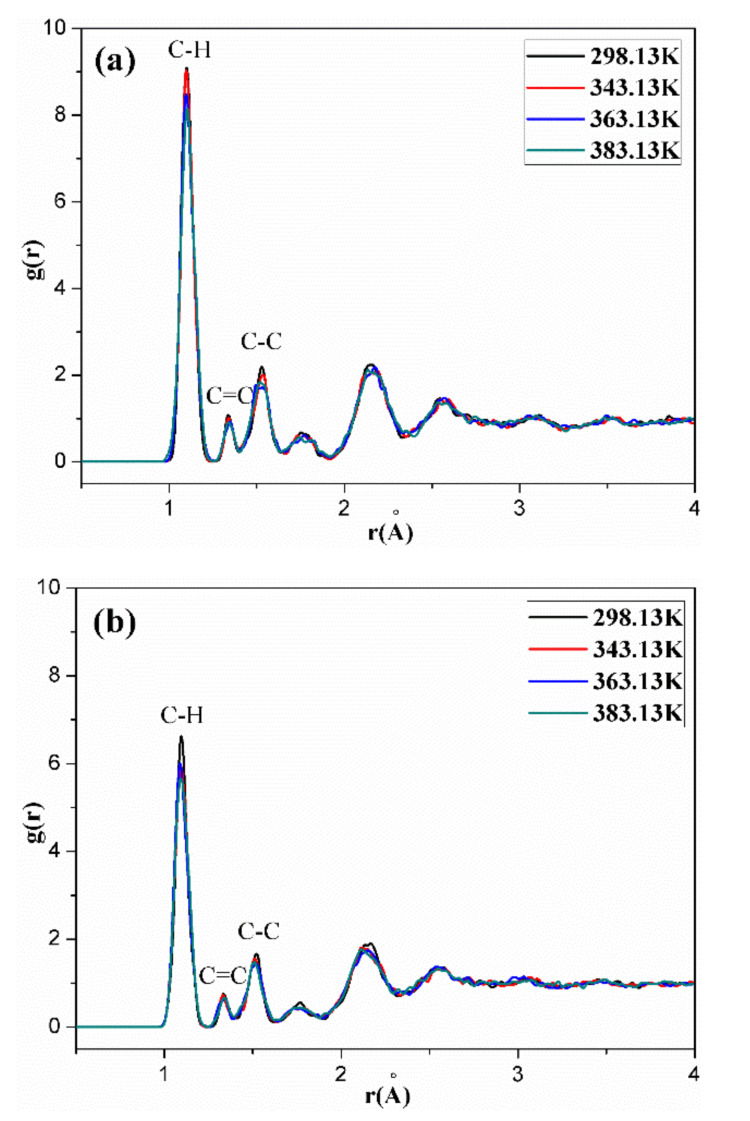
The molecule-molecule radial distribution functions (RDFs) of carbon atoms (C–C) in the NBR model in the uncompressed (**a**) and compressed (**b**) states.

**Figure 12:**
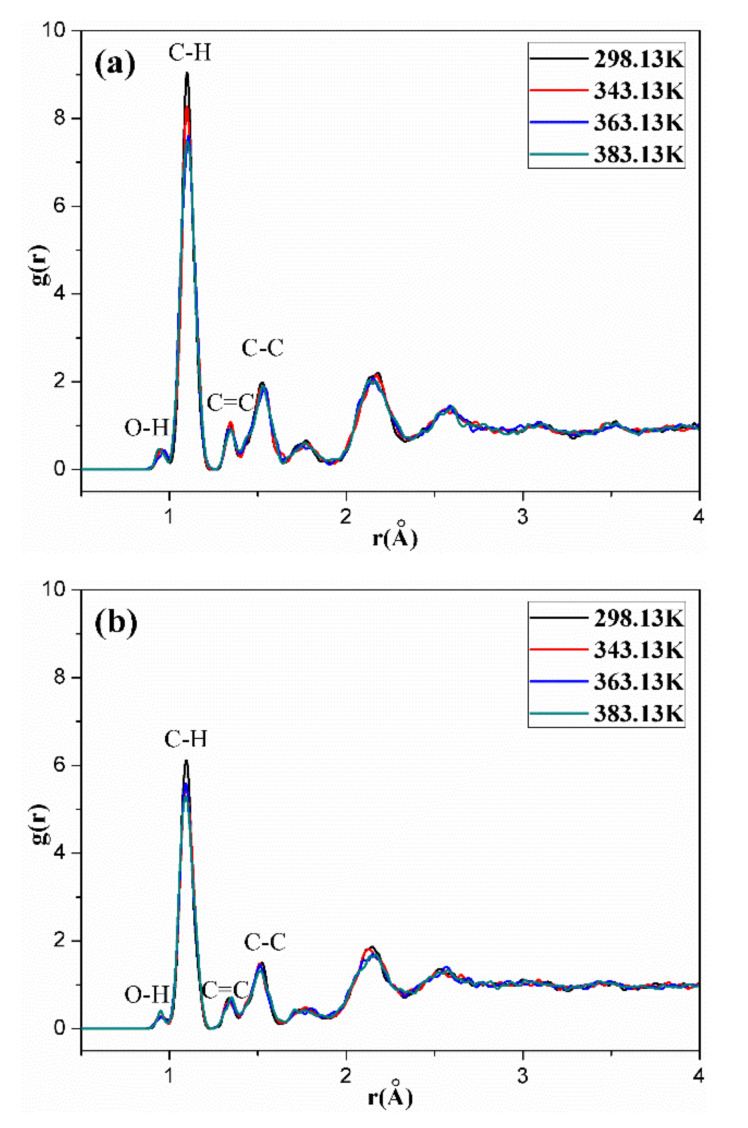
The radial distribution functions (RDFs) of carbon atoms (C–C) in the OH-NBR model in the uncompressed (**a**) and compressed (**b**) states.

**Figure 13:**
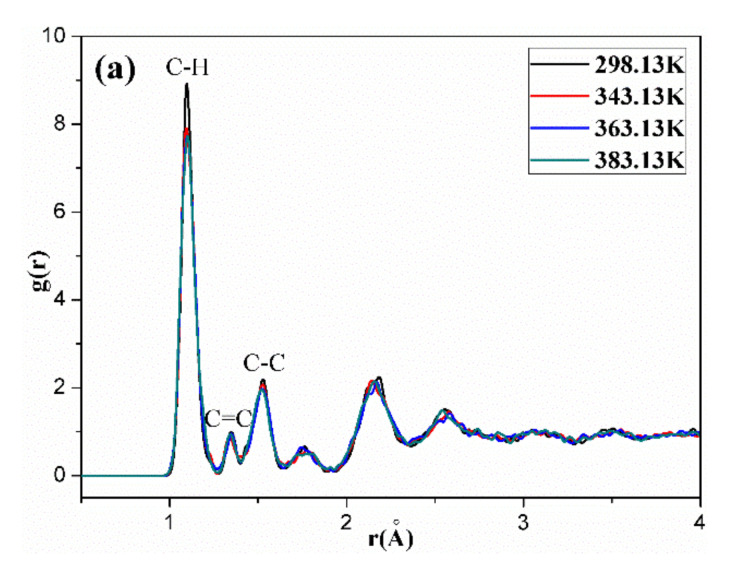

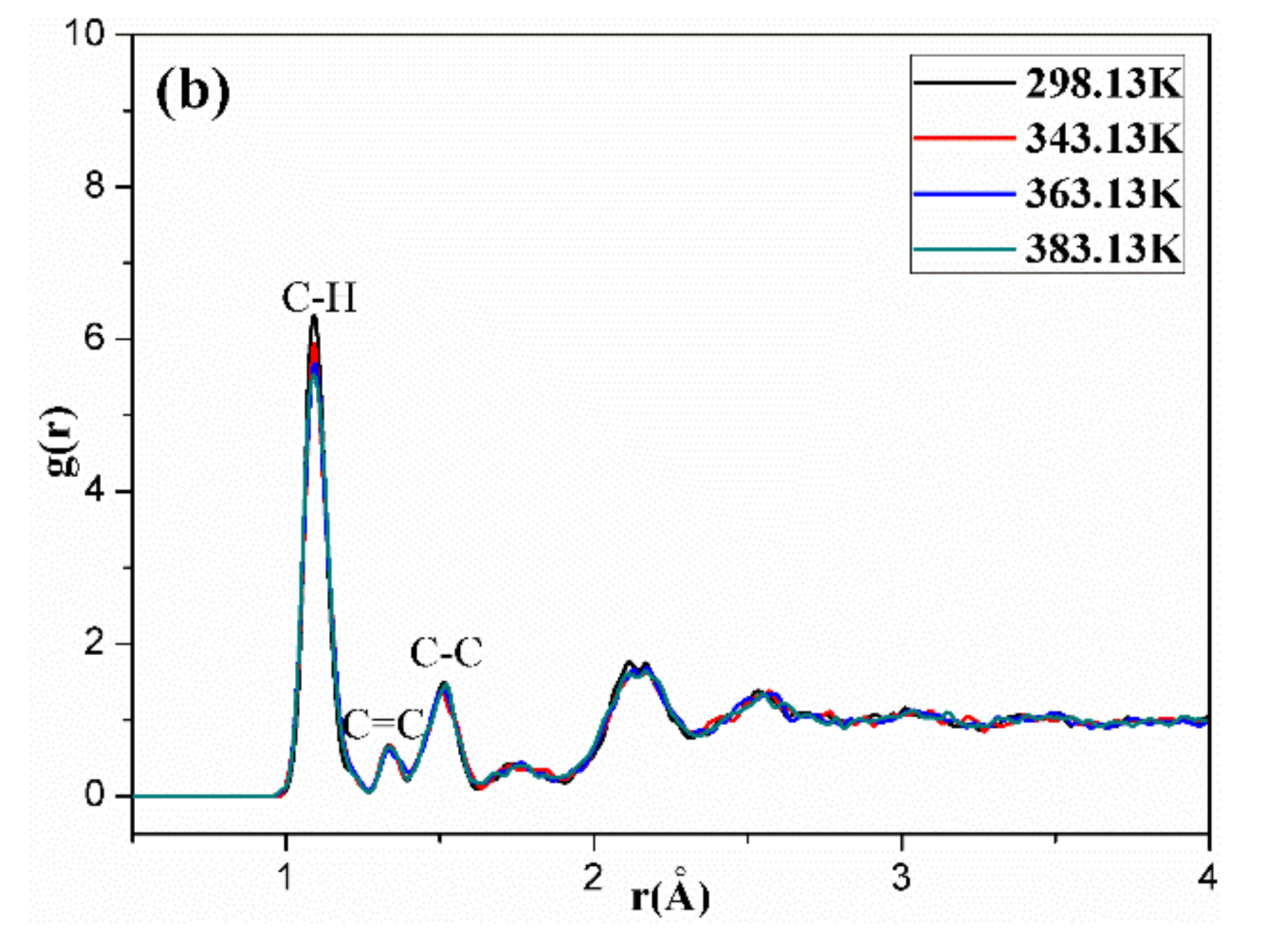
The radial distribution functions (RDFs) of carbon atoms (C–C) in the CO-NBR model in the uncompressed (**a**) and compressed (**b**) states.

**Figure 14:**
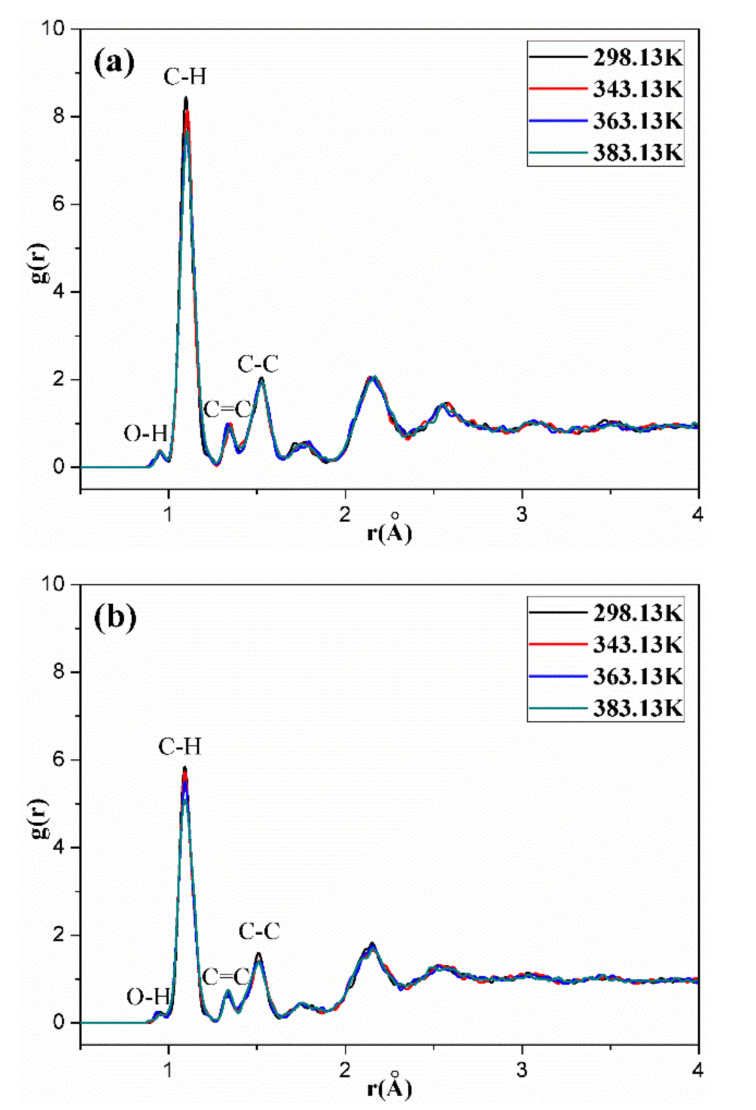
The radial distribution functions (RDFs) of carbon atoms (C–C) in the OH-CO-NBR model in the uncompressed (**a**) and compressed (**b**) states.

**Figure 15:**
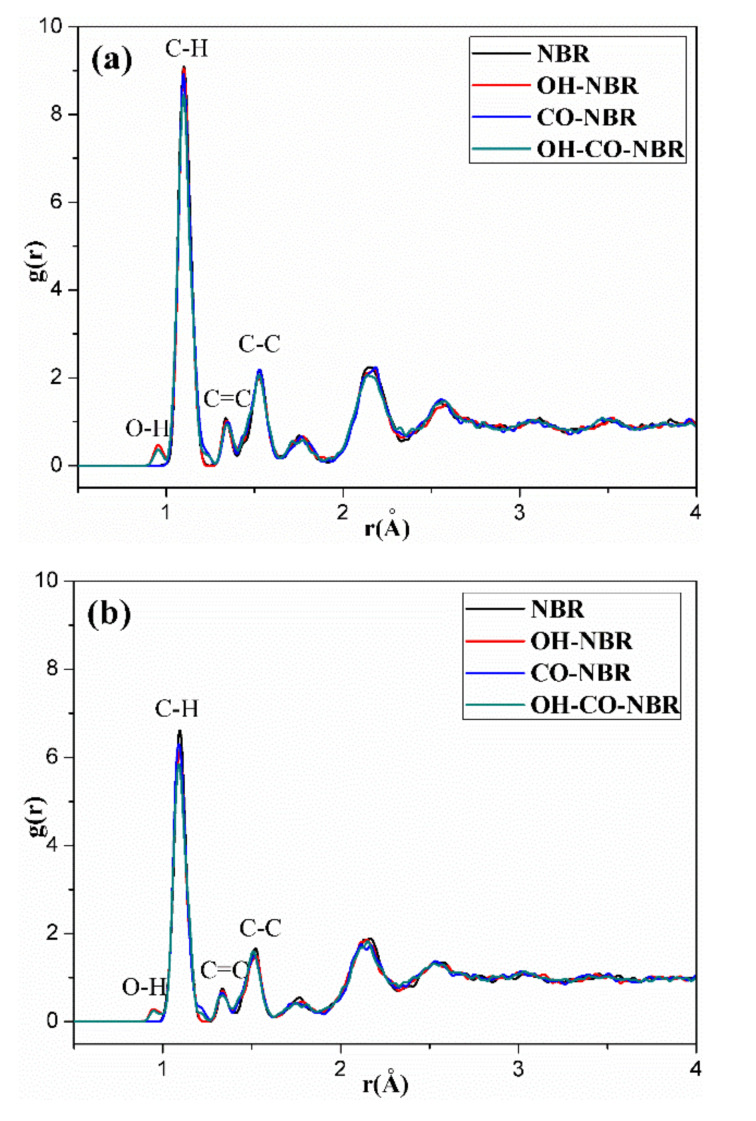
The radial distribution functions (RDFs) of carbon atoms (C–C) in the unaged and aged NBR model at 298.15K in the uncompressed (**a**) and compressed (**b**) states.

Figure 11, Figure 12, Figure 13, Figure 14 and Figure 15 show the RDFs of carbon atoms (C–C) in the unaged and aged NBR models under different conditions, respectively. The peaks at 1.10 Å, 1.33 Å and 1.53 Å can be assigned to the bond length of C–H, C=C and C–C, respectively [31,39]. The peak at 0.94 Å belongs to the bond length of O–H. Besides, when the distance is beyond 3.5 Å, no obvious peaks appear, implying that the models present long range disorder and the models are consistent with amorphous structure, which confirms the effectiveness of the constructed models. Additionally, the peak value of RDF decreased slightly with increase of temperature, indicating that the elevated temperature resulted in stronger mobility, an increase in flexibility and a decrease in the order of rubber chains. Furthermore, the peak value of RDF in the compressed state displayed a smaller value than that in the uncompressed state, implying that the compressive strain led to an increase in disorder and chain stacking. When the hydroxyl groups and carbonyl groups were used to modify the rubber molecular chains, it was found that the peak value of RDF showed a slight decrease in aged rubber models. This is mainly because the introduction of hydroxyl groups and carbonyl groups increased the intermolecular forces between molecular chains, and decreased the segment motion and flexibility of molecular chains.

### 3.5. Cohesive Energy Density (CED) and Solubility Parameter (δ)

As defined by Hildebrand and Scott [45], cohesive energy density (CED) can be used to characterize the strength of attractive interactions representing intermolecular force including van der Waals force and Coulomb force, and the solubility parameter (δ) is simply the square root of the cohesive energy density (CED).

Table 4 shows the cohesive energy density and solubility parameters of different oxidative aging models. The results indicate that the CED and solubility parameters of the aged model were higher than those of the unaged model, due to the stronger intermolecular interaction among molecular chains caused by the polar groups. Moreover, the CED and solubility parameters of the aged models conform to the order: OH-CO-NBR > OH-NBR > CO-NBR, due to the effect of strong polar interaction and the hydrogen bonds. When compressive strain was applied to the unaged and aged models, it was found that the CED of the rubber model in the compressed state was lower than that of the rubber model in the uncompressed state, implying that compressive strain weakened the intermolecular force. This is mainly because the compressive strain destroyed the strong polar interaction and hydrogen bond among the rubber molecular chains.

## 4. Conclusions

Aged NBR models were constructed by the introduction of hydroxyl groups and carbonyl groups in rubber molecular chains to investigate the influence of oxidative aging on the static and dynamic characteristics of nitrile rubber using MD simulation. The following conclusions can be drawn:

1. The MSD and RDF results show that elevated temperature promoted significantly the mobility of rubber chains, while the compressive strain displayed the opposite effect. The introduction of hydroxyl groups and carbonyl groups weakened the mobility of rubber molecular chains due to increases in polarity, intermolecular force, volume and rigidity of rubber chains.
2. Hydrogen bonding analysis results demonstrate that the interaction forces including the strong polar interaction and hydrogen bond among chains were intensified due to the introduction of hydroxyl groups and carbonyl groups. In the uncompressed state, high temperature destroyed the formation of H-bonds _(A)_ or O–H...N. The increase in cohesive energy density of the aged NBR models also reflected the formation of interaction forces.
3. The fractional free volume results indicate that the fractional free volume increased with increasing temperature, whereas the compressive strain and the introduction of oxidation functional groups decreased the fractional free volume.

## Figures and Tables

**Table 1:** Details of the simulation models.

Simulation Model	DP	*N*_chain_	*N*_atom_	$\rho_{MD}\;(g/{\mathrm{cm}}^{3})$	$\rho_{Exp}\;(g/{\mathrm{cm}}^{3})$	Cell Lengths (Å)
NBR model	50	5	2255	1.06	1.02	27.58
OH-NBR model	50	5	2295	1.09	-	27.71
CO-NBR model	50	5	2215	1.12	-	27.45
OH-CO-NBR model	50	5	2255	1.11	-	27.60

**Table 2:** The self-diffusion coefficients of unaged and aged NBR chains under different conditions.

Temperature/K	Self-Diffusion Coefficient (10^−7^ cm^2^ s^−1^)			
	NBR	OH-NBR	CO-NBR	OH-CO-NBR
298.15	1.83	1.41	1.09	0.94
383.15	4.66	4.14	3.98	2.72

**Table 3:** The types and number of H-bonds in aged NBR models.

Type of Hydrogen Bond	OH-NBR				OH-CO-NBR			
	Uncompresssed		Compressed		Uncompresssed		Compressed	
	298.15 K	383.15 K	298.15 K	383.15 K	298.15 K	383.15 K	298.15 K	383.15 K
A	26	20	22	26	18	13	14	16
B	6	4	5	5	2	2	2	2
C	0	0	0	0	2	5	2	2

**Table 4:** The cohesive energy density (CED) and solubility parameters of the unaged and aged NBR models.

Compression Set	Rubber Model	NBR	OH-NBR	CO-NBR	OH-CO-NBR
0	CED (10^8^ J/cm^−3^)	3.07	3.52	3.38	3.62
	δ (J/cm^−3^)^1/2^	17.51	18.77	18.41	19.02
30%	CED (10^8^ J/cm^−3^)	2.41	2.96	2.67	2.84
	δ (J/cm^−3^)^1/2^	15.53	17.20	16.35	16.85

## Data Availability

Data is contained within the article.
